# Baseline characteristics of rhegmatogenous retinal detachments meeting the PIVOT trial criteria in an eye referral center in Colombia: case series

**DOI:** 10.1186/s40942-025-00728-8

**Published:** 2025-09-24

**Authors:** Danny Salgado-Gómez, Luis C. Escaf, Omaira Díaz-Granados Gonzalez, Jorge Escobar-DiazGranados

**Affiliations:** 1 Clínica Oftalmológica del Caribe, Barranquilla, 080020 Colombia; 2 Department of Ophthalmology, Faculty of Health Sciences, Universidad Simón Bolívar, Barranquilla, Colombia

## Abstract

**Objective:**

This study aims to characterize the baseline profile and clinical outcomes of pneumatic retinopexy (PR) in patients fulfilling the Pneumatic Retinopexy versus Vitrectomy for the Management of Primary Rhegmatogenous Retinal Detachment Outcomes Randomized Trial (PIVOT) eligibility criteria at a tertiary referral center in Colombia.

**Methods:**

This retrospective consecutive case series included patients with primary rhegmatogenous retinal detachment (RRD) meeting PIVOT criteria between January 2021 and April 2024. The primary outcome was single-operation success (SOS) at 3 months, defined as complete retinal reattachment after one PR procedure, allowing for a supplementary gas injection within 21 days.

**Results:**

563 newly diagnosed primary RRD cases were reported, 143 (25.4%) were PIVOT-eligible and 105 underwent PR. The SOS rate was 85.7% (90/105). Baseline age, sex, lens status, and macular status were comparable between success and failure groups. Detachment extent > 2 quadrants correlated with an increased risk of failure (*p* = 0.045); however, this association should be interpreted cautiously given the limited number of events.

**Conclusions:**

Approximately one-quarter of primary RRD cases at our center met PIVOT criteria. In this cohort, PR yielded a high SOS rate and remains a valuable first-line surgical option in appropriately selected patients, particularly in settings with constrained resources.

## Background

Pneumatic retinopexy (PR) was introduced by Dominguez in 1985 and popularized by Hilton and Grizzard in 1986 as a minimally invasive alternative to scleral buckle and pars plana vitrectomy (PPV) for rhegmatogenous retinal detachment (RRD) repair [1–4]. This outpatient procedure involves intravitreal injection of an expandable gas bubble followed by retinopexy via laser photocoagulation or cryotherapy. PR offers advantages such as reduced surgical trauma, faster visual recovery, and lower costs compared with other surgical techniques [5–9].

The use of PR varies internationally. According to the Preference and Trends Survey of the American Society of Retina Specialists, 32.8% of US retinal surgeons perform PR as their primary surgical approach for selected RRDs, compared with 55.8% of non-US surgeons [10]. These differences may reflect variations in training, case selection, and perceptions of surgical outcomes.

Recent evidence, including the PIVOT trial, supports PR as a first-line treatment in carefully selected cases, demonstrating superior visual outcomes, less unintended retinal displacement, and fewer postoperative complications compared to PPV [2, 6, 7]. Specifically, PPV has shown a higher single-procedure anatomical reattachment rate than PR (93.2% vs. 80.8%), whereas PR has resulted in better visual acuity outcomes (79.9 letters vs. 75.0 letters) and fewer objectively measured metamorphopsias (18.8% less) [2]. The improved outcomes may result from a less invasive procedure that preserves retinal integrity and facilitates natural subretinal fluid reabsorption, reducing visual distortions such as metamorphopsia and micropsia [7].

Given global trends in surgical management and limited data from Latin America, this study aims to clarify the role of PR by characterizing RRD cases meeting PIVOT trial eligibility criteria in a tertiary ophthalmology hospital in Barranquilla, Colombia, and assessing the feasibility of PR in resource-constrained settings.

## Materials and methods

### Study design

This was a retrospective, consecutive case series conducted at a single center.

### Patients

Consecutive patients with RRD presenting to Clínica Oftalmológica del Caribe, a tertiary eye referral center in Barranquilla, Colombia, between January 2021 and April 2024, were evaluated. Recruitment was performed through a systematic search of electronic medical records using the ICD-10 code H33.

Eligible patients met the criteria of the Pneumatic Retinopexy versus Vitrectomy for the Management of Primary Rhegmatogenous Retinal Detachment Outcomes Randomized Trial (PIVOT), as defined by Hillier et al. [2]: a single retinal break or a group of breaks not exceeding one clock hour (30°) in detached retina; all breaks in detached retina located above the 8 and 4 o’clock meridians; and any breaks or lattice degeneration in attached retina permitted regardless of location.

Patients were excluded from the study if they presented inferior retinal breaks in detached retina, significant media opacities (e.g., dense vitreous hemorrhage or cataract precluding adequate retinal examination), proliferative vitreoretinopathy grade B or higher, history of retinal detachment or pars plana vitrectomy in the index eye, ocular trauma, concurrent ocular infection, age under 18 years, mental incapacity, or inability to maintain prescribed postoperative head positioning. Additionally, patients presenting significant posterior capsular opacification, capsular phimosis, or poor mydriasis were also excluded. Specific strategies were implemented to address potential visualization challenges, such as pharmacologic reinforcement of mydriasis and scleral depression.

### Ophthalmic examination

Baseline assessment included best-corrected visual acuity (BCVA), slit-lamp biomicroscopy, and dilated fundus examination with indirect ophthalmoscopy, all performed by the operating surgeon.

### Surgical procedure

All procedures were performed in the operating room under topical anesthesia with patients supine position. Standard aseptic preparation with 5% povidone–iodine for the skin and 10% for the ocular surface, and lid speculum insertion were followed by a paracentesis of approximately 0.3 mL aqueous humor via a 30-gauge needle at the limbus in the superior nasal quadrant.

A 100% concentration of either sulfur hexafluoride (SF₆; 0.5–0.6 mL) or perfluoropropane (C₃F₈; 0.3–0.4 mL) was injected into the vitreous cavity through a 30-gauge needle placed 3.5–4.0 mm posterior to the limbus. Reflux prevention and immediate confirmation of central retinal artery perfusion and hand motion vision were ensured.

Retinal breaks were treated 24–48 h post-injection with a 532 nm argon green laser delivered via indirect ophthalmoscope or slit-lamp, depending on tear location and patient cooperation. Postoperative positioning was indicated according to break location. Follow-up visits were scheduled at 1 week, 3 weeks, and 3 months post-procedure.

### Outcomes and bias mitigation

Primary outcome was single-operation success (SOS) at 3 months, defined as complete anatomical retinal reattachment after a single PR, allowing one additional gas injection within 21 days.

Secondary outcomes included, baseline predictors of SOS, and time intervals from symptom onset to consultation and surgery.

Potential biases were mitigated by inclusion of all consecutive PIVOT-eligible cases (selection bias), use of standardized ICD-10 codes and structured data collection (information bias), and focusing analysis on SOS in PR-treated patients without non-randomized comparisons (confounding).

### Statistical analysis

Data were analyzed using IBM SPSS Statistics v29. Continuous variables were assessed for normality and compared using appropriate parametric or non-parametric tests. Categorical variables were compared using Chi-square or Fisher’s exact tests. A p-value < 0.05 was considered statistically significant.

## Results

During the study period, 1,469 medical records coded for retinal detachment were reviewed. 563 cases of new-onset RRD remained for analysis after excluding 906 cases that corresponded to chronic RRD, tractional or serous retinal detachments, retinal detachment secondary to trauma, or patients with a prior history of RD who retained the ICD-10 code despite having undergone previous surgical treatment.

**Fig. 1:**
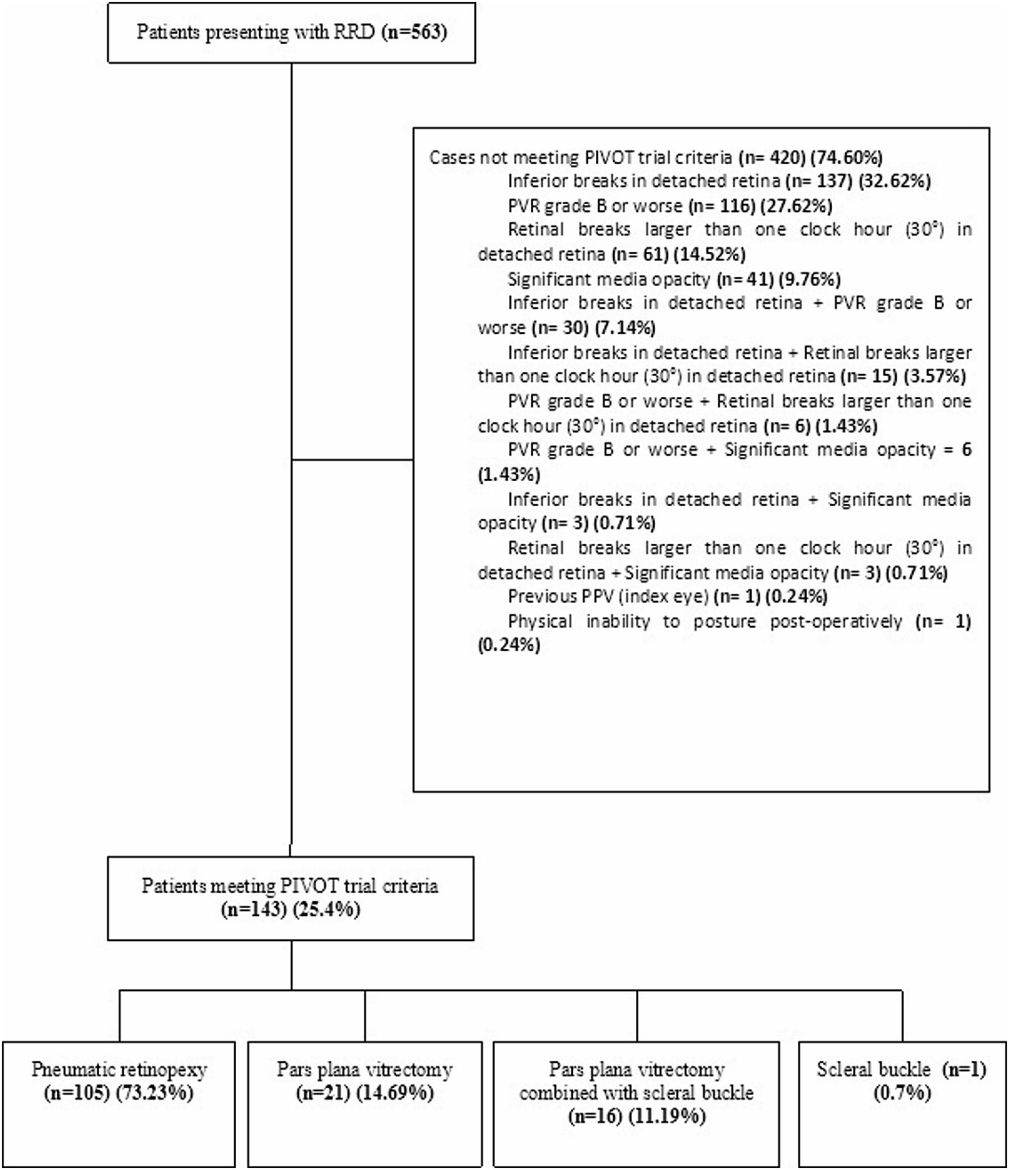
Flow diagram representing the distribution of rhegmatogenous retinal detachments (RRD) that met the PIVOT criteria

Of the 563 new-onset RRD cases, 143 (25.4%) met PIVOT trial criteria and were included, while 420 (74.6%) did not meet criteria and were excluded (Fig. 1). Among included cases, 105 (73.4%) underwent PR, 16 (11.2%) underwent primary PPV combined with scleral buckle, 21 (14.7%) received PPV alone, and 1 (0.7%) underwent scleral buckle alone (Fig. 1). The SOS rate for PR was 85.7% (90/105), with 15 eyes (14.3%) requiring subsequent PPV.

Baseline characteristics including mean age (57.1 vs. 57.8 years; *p* = 0.79), sex distribution (*p* = 0.57), and lens status (phakic 81.1%, pseudophakic 17.8%, aphakic 1.1%; *p* = 0.36) were similar between SOS and failure groups. Macula-on detachments were present in 35.6% of SOS and 26.7% of failure cases. Mild vitreous hemorrhage was noted in 6 patients (5 SOS, 1 failure). Median time from symptom onset to procedure did not differ significantly (SOS: 10 days [IQR 3–30.25], failure: 7 days [IQR 4–24], *p* = 0.81).

Laser retinopexy, an intrinsic PR component, was performed in 95.2% of cases. Additional gas injection was administered in 15.2%, and cryopexy was performed in 2.9% under topical anesthesia (Proparacaine Hydrochloride 0.5%) when laser treatment was not feasible due to tear location. (Table 1).

**Table 1:** Details of patients with rhegmatogenous retinal detachments who Met PIVOT criteria and were treated by pneumatic retinopexy

Variable	*n* (%) *n* = 105
Patients with preoperative laser treatment	1 (0.9)
Cryopexy	3 (2.8)
Patients with postoperative laser retinopexy	100 (95.2)
Patients with additional gas injection*	16 (15.2)

*Number of patients requiring additional gas injections within 21 days after the operative procedure. Any additional gas injection beyond 21 days was considered a failure of primary treatment

Predictors of SOS identified by univariate analysis included macula-on presentation and absence of vitreous hemorrhage; lens status, age, sex, and symptom duration were not significantly associated (Table 2). Detachment extent > 2 quadrants correlated with an increased risk of failure (*p* = 0.045).

**Table 2:** Baseline characteristics of the 105 eyes meeting PIVOT trial criteria that underwent pneumatic retinopexy

Variable	Pneumatic Retinopexy Success (*n* = 90)	Pneumatic Retinopexy Failure (*n* = 15)	*P* Value
Age (yrs), mean (SD)	57.11 (9.96)	57.80 (6.27)	0.796
Gender, no. (%)			0.576
Male	49 (54.44)	7 (46.67)	
Female	41 (45.56)	8 (53.33)	
Preoperative lens status, no. (%)			0.357
Phakic	73 (81.11)	10 (66.67)	
Pseudophakic	16 (17.78)	5 (33.33)	
Aphakic	1 (1.11)	0 (0.00)	
Macular status, no. (%)			0.502
Macula on	32 (35.56)	4 (26.67)	
Macula off (including split)	58 (64.44)	11 (73.33)	
Detachment size, no. (%)			0.045
≤2 quadrants	8 (8.89)	4 (26.67)	
>2 quadrants	82 (91.11)	11 (73.33)	
Category based on location of the lowest break, no. (%)			0.440
Category 1 (12-o’clock meridian)	9 (10.00)	1 (6.67)	
Category 2 (11- or 1-o’clock meridians)	41 (45.56)	5 (33.33)	
Category 3 (10- or 2-o’clock meridians)	26 (28.89)	8 (53.33)	
Category 4 (9- or 3-o’clock meridians)	12 (13.33)	1 (6.67)	
Category 5 (8- or 4-o’clock meridians)	2 (2.22)	0 (0.00)	
Presence of retinal breaks in attached retina, no. (%)	2 (2.22)	0 (0.00)	0.560
Lattice degeneration in attached retina, no. (%)	6 (6.67)	1 (6.67)	1.00
Vitreous hemorrhage, no. (%)	5 (5.56)	1 (6.67)	0.864
Days from symptom onset to surgery, median (IQR)	10 (3–30.25)	7 (4–24)	0.812
Days from first consult to surgery, median (IQR)	0 (0–1)	0 (0–0)	0.079

SD = standard deviation

All 15 failed PR cases underwent PPV; in 4 cases, PPV was combined with scleral buckle due to extensive peripheral pathology.

## Discussion

Our study demonstrated that 25.4% of patients presenting with RRD at a tertiary center met PIVOT trial criteria and were candidates for PR as a first-line treatment. While PPV offers a higher primary reattachment rate, PR confers advantages including less invasiveness, minimal retinal displacement, improved visual function, fewer refractive changes, reduced cataract formation, and shorter recovery [2, 5, 8, 11]. Additionally, PR’s lower costs may benefit healthcare systems with limited resources [8, 9].

The lower eligibility proportion compared to Hillier et al. 50% [2, 6] could be explained by our high prevalence of advanced PVR: 27.6% grade B or worse, including 14.0% grade C or worse. Excluding cases with PVR ≥ grade B increases eligibility to 35.1%, narrowing differences. Other factors include strict exclusion of media opacities (9.8%), large or inferior tears (32.6% and 14.5%), and local referral patterns leading to more complex RRDs.

The study’s SOS rate of 85.7% aligns with a meta-analysis reporting 74.4% average SOS [12] and Yannuzzi et al.’s 68.5% [13]. While some series report higher rates (82%) [14, 23], a multicenter study showed 67% SOS by vitreoretinal fellows, improving to 86.2% after > 15 cases [14], highlighting the importance of surgical experience to improve success rates.

Lens status was not statistically significant, contrasting prior findings where pseudophakia predicted failure [15–17]. This may be due to careful patient selection excluding eyes with posterior capsule opacification or poor mydriasis and use of enhanced visualization techniques as scleral depression. Single-operation success for detachments involving > 2 quadrants was observed, though literature reports poorer outcomes with detachments > 4 quadrants [18–20].

Patients excluded from PR eligibility mostly presented with inferior tears and advanced PVR, both major failure predictors [21, 22]. PVR’s association with chronicity, inflammation, and other factors underscores the importance of thorough preoperative assessment [22].

Symptom-to-treatment timing did not significantly differ between groups, though the short median delay suggests timing might influence outcomes and warrants further study, especially in resource-limited settings where PR offers a viable option [2, 9].

We acknowledge the inherent limitations of our study, including its retrospective nature and data collection from secondary sources. This methodology also limits the ability to establish causality. It is important to note that sampling was convenience-based according to the availability of records in databases, potentially leading to selection and information bias. The reliance on data from electronic medical records may introduce inaccuracies or missing information. Additionally, the lack of a comparative group undergoing alternative treatments limits the ability to draw definitive conclusions about the efficacy of this intervention. Moreover, there was no standardized protocol for the intervention as it was at the discretion of the surgeon performing initial evaluation. On the other hand, the strength of this study lies in its population base. Although it was conducted at a single reference center in Barranquilla, this institution serves patients across all socioeconomic strata and provides the most extensive ophthalmologic coverage on the Colombian Caribbean coast. This broad coverage, combined with a robust methodology that included a well-defined study population, clear inclusion and exclusion criteria, and the systematic collection of demographic, clinical, and follow-up variables, enabled a detailed and representative characterization of RRD cases in our setting. These elements offer a comprehensive understanding of the local epidemiology and clinical profile of patients, providing valuable information to guide clinical decision-making and healthcare planning in the region.

## Conclusion

This study supports pneumatic retinopexy as an effective first-line treatment with favorable anatomical outcomes in selected patients with RRD meeting PIVOT trial criteria. Our findings reaffirm PR’s value in clinical settings where resources may be limited, emphasizing the importance of careful case selection and suggesting that PR remains a safe, cost-effective, and minimally invasive alternative to vitrectomy in eligible patients.

These insights also contribute to a better understanding of RRD characteristics and management in Latin America and support the incorporation of PR into evidence-based treatment algorithms tailored to local healthcare environments.

## Data Availability

No datasets were generated or analysed during the current study.

## Abbreviations

C3F8: Octafluoropropane
IQR: Interquartile range
PIVOT: Pneumatic Retinopexy versus Vitrectomy for the Management of Primary Rhegmatogenous Retinal Detachment
PPV: Pars plana vitrectomy
PR: Pneumatic retinopexy
PVR: Proliferative Vitreoretinopathy
RRD: Rhegmatogenous retinal detachment
SF6: Sulfur hexafluoride
SOS: Single operation success
SD: Standard deviation
